# Gastritis1Read at the Tenth Annual meeting of the Illinois Veterinary Medical and Surgical Association, at Decatur, Illinois, on January 12, 1899.

**Published:** 1899-07

**Authors:** F. Glassbrenner

**Affiliations:** North Alton, Ill.


### GASTRITIS [1]

By F. Glassbrenner, V.S.,
NORTH ALTON, ILL.

Gastritis, or inflammation of the mucous membrane lining the stomach, may be acute or subacute. Acute gastritis is generally caused by some highly irritating substances being taken into the stomach, such as giving poisons in too large doses, or when not sufficiently diluted. The subacute form is more frequently seen, and if not properly treated may extend into the bowels, and we have gastro-enteritis and a dangerous disorder to contend with.

It is remarkable, however, all things considered, how seldom

[1] Read at the Tenth Annual meeting of the Illinois Veterinary Medical and Surgical Association, at Decatur, Illinois, on January 12, 1899.

the stomach is affected with inflammation. It is not only a receptacle for the food, but is to be regarded as a glandular organ furnishing daily a large quantity of an important secretion—the gastric juice. So when the the inner surface of the stomach is inflamed we have to deal with a mixed condition that is partly a catarrhal inflammation.

Symptoms. If the gastritis is caused by poison the symptoms will largely depend upon the variety of poison which has been taken, but in the most cases which I have seen there is a strange sort of drowsiness. The animal does not lie down to sleep, but will rest himself against anything he can, and hang his head nearly to the ground. Colicky pains will be present and nearly always intense thirst. Could the animal speak, he would complain of a burning sensation in his stomach. The pulse is very feeble, at times it can hardly be felt. There will be some fever, the temperature will rise and fall, but will increase in intensity as the disease progresses. He will sweat freely in most cases and the saliva flows from the mouth. This last will only occur when the throat is inflamed so that he cannot swallow. If the gastritis is due to poisoning by lead, there will be a rough, staring coat, a contracted or tucked-up appearance of the abdomen, and some fever. As the disease progresses partial paralysis will come on at intervals. The breathing will be difficult, which I think is due to a partially paralyzed state of the respiratory apparatus. There will also be a bluish line along the margin of the gums. If the gastritis, or gastro-enteritis as the case may be, is caused by arsenic, there will be greater abdominal pain than in a case of lead-poisoning; this is indicated by the horse looking eagerly at his flanks. There will also be purging, hurried breathing, and the saliva flows from the mouth; the animal will stagger, and finally it becomes paralyzed in the hind extremities and death follows. The symptoms are almost the same if the poisoning is by corrosive sublimate.

Treatment. To map out the proper treatment for gastritis is difficult, because the treatment will depend a good deal upon what has caused the trouble. It is well to be careful in diagnosing the case; in fact, we should be more particular than the human practitioner, as our patients cannot speak and tell us where the seat of the pain is. We must not think that every animal that is in pain has the colic. Though gastritis is not a common disorder, still I think the stomach is occasionally the subject of inflammation when we are not aware of the fact, and a case of gastritis is sometimes treated as a case of colic. This would not be so bad if the medicine used were not of an irritating nature, but unfortunately many of the remedies used by the inexperienced for colic add fuel to the fire where there is any inflammation, and a fatal case of gastro-enteritis is developed from a slight case of gastritis.

In cases of poisoning, and when the case is seen in time before the poison has entered the circulation, the stomach should be emptied and washed out by means of the stomach-pump, and in the case of animals capable of the act of vomiting give an emetic. In cases of poisoning by arsenic give, as soon as possible, peroxide of iron at intervals of ten minutes, until a quantity twelve times greater than that of the poison has been taken. When a case of acute gastritis is known to be caused by lead give from forty to sixty drops of sulphuric acid well diluted. If the gastritis is chronic, due to the continued taking into the stomach of small quantities of lead, give a brisk but mild physic. This should be followed by drachm doses of iodide of potassium. When there is much pain give opiates. In this case I think morphine is best, repeated as often as necessary. In all cases demulcent drinks, oils, white of eggs, anything that will soothe and protect the inflamed membrane, should be used; counter-irritation over the stomach and bowels will be of some service.

Post-mortem examination in case of lead-poisoning will show the lining of the stomach and sometimes that of the intestines to be darker in places; the liver and kidneys also have a bluish tinge. In poisoning by arsenic the carcass when opened will give out large quantities of fetid gas; the lungs are congested, and their mucous membrane reddened. These appearances will differ with the severity and duration of the case.

